# Surgical disaster following hernia mesh infection and erroneous treatment strategy: A case report

**DOI:** 10.1016/j.ijscr.2020.04.028

**Published:** 2020-05-08

**Authors:** Arnolds Jezupovs

**Affiliations:** a Clinic of Surgical Infections, Riga East University Hospital, Riga, Latvia; b Faculty of Medicine, University of Latvia, Riga, Latvia

**Keywords:** Ventral hernia, Mesh infection, Enteroatmospheric fistula, Surgical errors, Case report

## Abstract

**Introduction:**

The aim of the current case presentation is to demonstrate the calamity of hernia mesh infection arising from an erroneous surgical strategy.

**Presentation of case:**

A patient with a recurrent gigantic ventral hernia and chronic hernia mesh infection is presented. 56 months and five surgeries were necessary to resolve the hernia mesh infection, and 7 months were required to deal with the complications following mesh explantation. During the last hospitalization, 18 surgical interventions under general anesthesia, 12 radiologic, 13 microbiological and 41 laboratory examinations were performed. Seven antibiotics were prescribed for 112 days in total.

**Discussion:**

It is challenging for an ordinary surgeon to be up to speed with the latest evidence-based practices if dealing with a surgical domain not practiced regularly. Tactical errors play as big a role as errors in surgical technique. A surgical strategy varying between error and accuracy can catalyze a chain reaction of complications and surgical errors, finally resulting in life-threatening complications.

**Conclusion:**

The case report demonstrates the impact of an erroneous surgical strategy on the treatment process and highlights the difficulties related to hernia mesh infection.

## Highlights

• Hernia mesh infection is easier to prevent than to cure.
• A late infected hernia mesh explantation is more challenging than an early one.
• Hernia mesh fragmentation following partial explantation burdens its complete removal.
• An incorrect surgical strategy could catalyze a chain reaction of complications.

## 1 Introduction

The risk of making the wrong decision on the treatment strategy of a surgical pathology is higher nowadays than a century ago. A fast development of technology has resulted in a number of surgical approaches and devices available for abdominal hernia repair, thus challenging decision making in certain clinical situations. Hernia mesh infection is a “surgical disaster” [1] with a devastating effect on all involved. The current case presentation is a notable example of the calamity arising from an incorrect surgical strategy.

This work has been reported in line with the SCARE criteria [2].

## 2 Presentation of case

A 42-year-old man (BMI 37) with a gigantic recurrent ventral hernia and chronic hernia mesh infection was hospitalized with complaints of a large volume of purulent discharge from newly-appeared abdominal skin openings. The patient`s medical history was complex and begun 10 years ago when the patient fell from a height of 13 m. Midline laparotomy was performed four times because of peritonitis and resulted in the development of a large incisional hernia, the treatment of which consisted of four hospitalizations in the previous 56 months.

### 2.1 Hospitalization 1

The patient underwent the repair of a large (the defect width of 12.2 cm and height of 18.8 cm on CT scan) incisional hernia by an open bridged onlay mesh technique, applying the *Parietex Composite®* mesh of unknown size fixed to the external oblique muscle aponeurosis, in another surgical facility by another surgeon 56 months ago. Resection of 1.5 m of the small intestine, the great omentum and appendectomy was done simultaneously with the purpose of reducing intra-abdominal pressure. The postoperative course was complicated by a spontaneous drainage of seroma and an open wound at the disruption site. The patient was discharged with recommendations to continue regular dressings on an outpatient basis.

### 2.2 Hospitalization 2

The patient was readmitted to the previous surgical facility two weeks after discharge because of a non-healing wound. A CT scan confirmed an early recurrence of hernia (the defect width of 14.7 cm and height of 19 cm). The examination revealed serous discharge and a fibrin coat around the mesh that was considered as a normal aseptic inflammatory reaction to the mesh. Treatment by applying the vacuum assisted closure (VAC) system was initiated, and improvement was stated since granulation tissue had appeared and the wound size had reduced twice. The patient was discharged with recommendations to finish treatment at an outpatient setting.

### 2.3 Hospitalization 3

The patient was re-hospitalized to the article author`s hospital eight months after the previous discharge complaining of purulent discharge from the skin opening and surgical wound.

A large recurrent irreducible ventral hernia and hernia mesh infection were confirmed. Two attempts of partial mesh removal were not effective because the infection continued. Complete mesh removal was stated after the third attempt. The patient was discharged with a secondary healing wound and recommendations to return for skin grafting after reaching the granulation phase.

### 2.4 Hospitalization 4

The patient appeared six weeks later with complaints of a new purulent skin opening approximately 5 cm away from the well-granulated wound. Sinus tract open revision was abandoned and mesh finding failed because of the high risk of damaging the visceral organs in a dense tissue conglomerate. A small intestine loop eviscerated on the 2^nd^ postoperative day and was treated conservatively. Constant sinus tract formation was recommended on discharge.

**Fig. 1:**
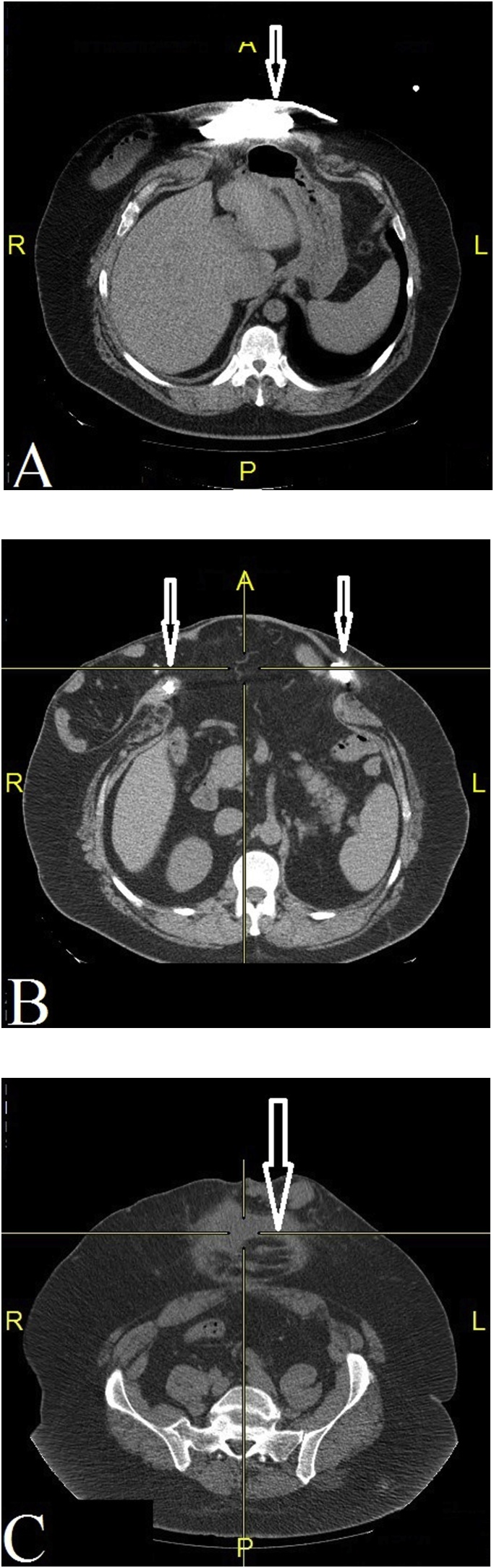
(A) The arrow shows the upper horseshoe abscess cavity at epigastrium. (B) Arrows show abscess cavities interconnected fistula tracts at mesogastrium. (C) The arrow shows the lower horseshoe abscess cavity at hypogastrium.

Now on the 5^th^ admission (six months after hospitalization 4) CT fistulography revealed a gigantic ventral hernia (the defect width of 19 cm and height of 26 cm) with abdominal domain loss and two horseshoe abscess cavities interconnected by two fistula tracts passed through both lateral surfaces of the hernia sac penetrating them on the right side (Fig. 1)

In order to fight the mesh infection definitely, the decision of the infected mesh explantation was made irrespective of the high risk for visceral and infectious complications.

**Fig. 2:**
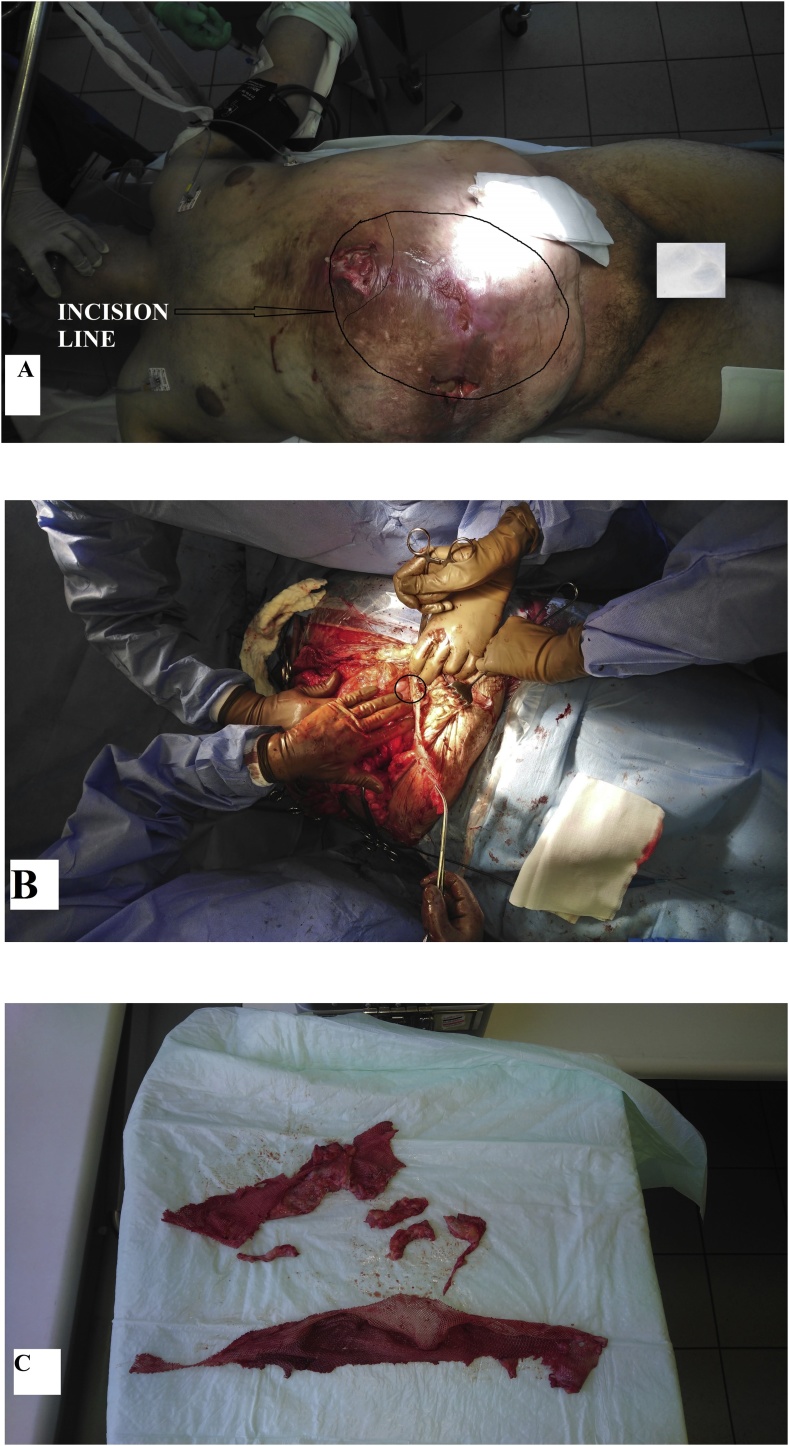
(A) A view of the abdomen before mesh removal. The lines indicate incision direction. (B) Separation of the infected mesh from the sigmoid colon. (C) Fragments of the explanted infected polyester mesh.

A herniolaparotomy was started in the epigastrium and continued by incisions over the fistula tracts down to the symphysis (Fig. 2A). Mesh explantation (Fig. 2C) was challenging due to the small intestines and the sigmoid colon being fixed densely to the mesh (Fig. 2B). Covering the sigmoid colon by any tissue was impossible because ischemic skin between the incisions had also been excised. Moreover, the operation field was grossly contaminated. The surgery ended by the abdominal VAC system application of 50 mmHg pressure over the wound.

**Fig. 3:**
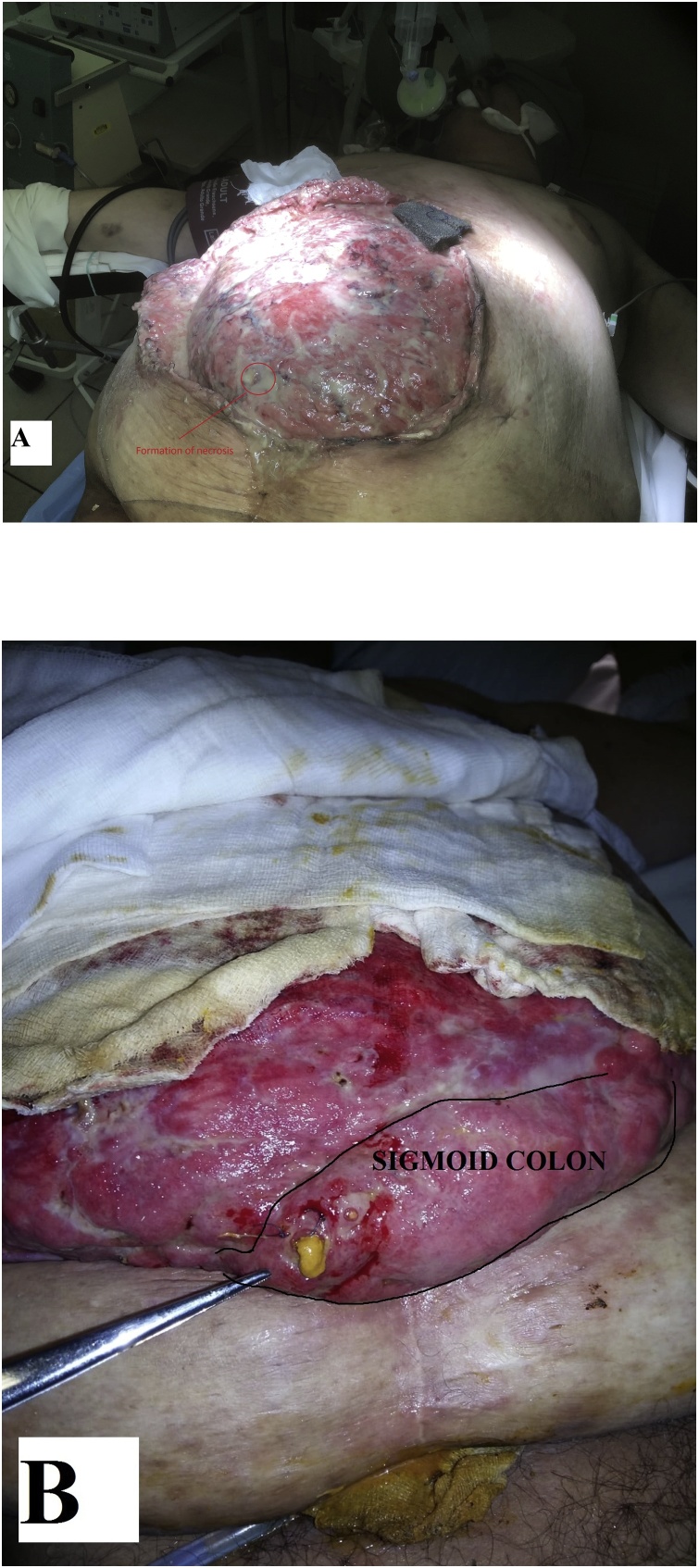
(A) A view of the abdominal wound after VAC system removal on the 6th day after mesh explantation. (B) Sigmoatmospheric fistula.

The VAC system was removed on the 6^th^ postoperative day. The wound looked infected with multiple abscesses in subcutaneous pockets. A formation of the sigmoid colon wall necrosis was noted (Fig. 3A). The patient was put on everyday dressings under general anesthesia. A 1–2 mm sigmoatmospheric fistula was found during the dressing on the 10^th^ postoperative day and was stitched. A second attempt at the recurrent fistula (Fig. 3B) closure was made three days later, strengthening the suture line by the *Tachosil®* sponge, but also failed.

The wound care was provided with regular dressings many times per day because of the inability to fix a stoma bag.

**Fig. 4:**
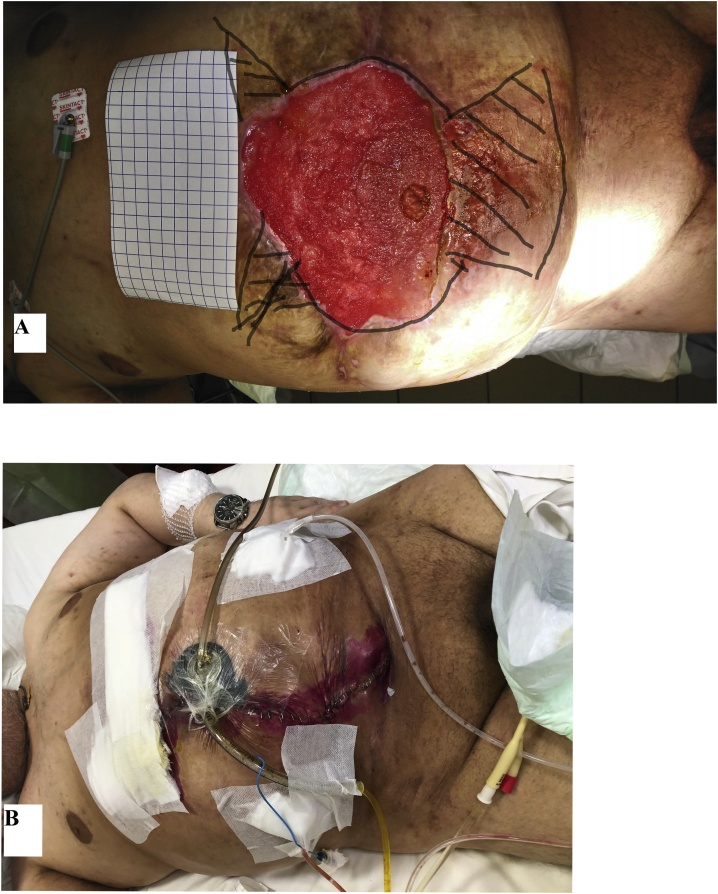
(A) A view before defect closure by local tissue flaps. (B) A view after defect closure on the 1st postoperative day.

In order to provide a faster stoma bag fixation, an attempt to close the skin defect by local flaps was made two months later (Fig. 4). The sigmoid colon was intubated, accompanied by the VAC system around the tube with the aim to protect the wound from colonic spillage.

**Fig. 5:**
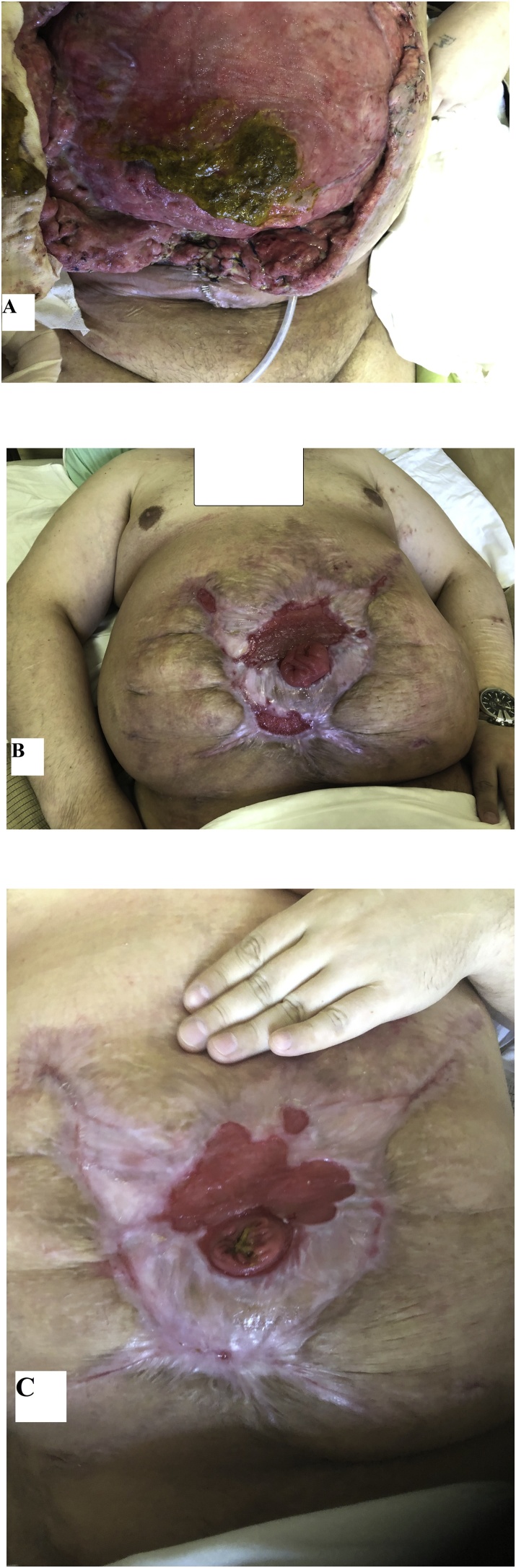
(A) A view on the 11th day, (B) on the 84th day and (C) on the 126th day after the failed surgery by local tissue flaps.

The procedure failed, and a wide spread of feces was found beneath the rotated flaps on the 3^rd^ day postoperatively. The defect appeared bigger than after mesh explantation (Fig. 5A). The patient was seriously ill for 10 days. An uncontrolled body temperature up to 40.3 °C all around 24 h was fixed. The patient`s condition improved gradually. Any surgical attempts to close the defect were not applied anymore. The defect healed spontaneously and the patient was discharged from hospital five months later when a hermetic fixation of the stoma bag was achieved.

To recap, 56 months and five surgeries were necessary to resolve the hernia mesh infection, and 7 months were spent on dealing with the mesh removal complications. The patient spent 7.5 months in hospital during the 5^th^ hospitalization. During that hospitalization, 18 surgical interventions under general anesthesia, 12 radiologic, 13 microbiological and 41 laboratory examinations were performed. Seven antibiotics were prescribed for 112 days in total. Many erroneous actions were noted retrospectively (Table 1). Finally, the patient had no clear benefit from the treatment since sigmostoma was obtained instead of the mesh infection, and the gigantic hernia was not repaired.

**Table 1:** The main erroneous actions during the treatment process.

Erroneous actions	Comment
Hernia repair by an open bridged mesh onlay technique (made by another surgeon)	This technique has an unacceptably high recurrence rate [3].
	A component separation or the peritoneal flap technique with mesh augmentation is a better choice [4,5].
Resection of the small intestine and appendectomy with the aim to reduce intra-abdominal pressure (made by another surgeon)	Less brutal methods exist to reduce intra-abdominal pressure [6].
	Associated enterotomy is an independent risk factor for mesh infection [7].
Unnoticed mesh infection and no mesh removal performed (made by another surgeon)	Mesh salvage was not reasonable because of early hernia recurrence. Early mesh removal would be easier to perform versus late when dense scar tissue has formed [8].
Partial removal of the infected mesh (made by the author of the article)	Incomplete removal of the infected mesh results in a chronic long lasting infection [9] and hinders every following attempt.
A late wound revision after applying the vacuum assisted closure after definitive mesh removal (made by the author of the article)	A six day interval was too long in case of a grossly contaminated wound. Every day wound control is recommended till granulation tissue appears.
Stitching of sigmoatmospheric fistula (made by the author of the article)	Closure of an enteroatmospheric fistula by sutures is almost impossible and resulted in a bigger hole.
Attempt to close the defect by local flaps in case of a sigmoatmospheric fistula (made by the author of the article)	It is impossible to provide a hermetic isolation of an enteroatmospheric fistula and an attempt to do it could result in a catastrophic outcome.

## 3 Discussion

Technological developments have allowed extending the range of surgical activities in all domains of surgery. A surgeon would not try repairing a gigantic ventral hernia with domain loss in the era before hernia implant invention. But the implant alone is insufficient for a successful outcome as confirmed by this case report. Furthermore, any medical device per se could cause an adverse event. The true incidence of such events, especially those arising from incorrect device application, is unknown because they are significantly underreported [10]. Medical errors would be the third leading cause of death in the United States if it were categorized as a disease [11].

Chronically infected mesh should be removed completely to resolve the infection. But the challenge lies in the difficulty of determining the extent of the infection, especially late after implantation when dense scar tissue has formed around the implant. Furthermore, infected areas could drain through the narrow convoluted sinus tract with a low volume output and could be easily missed during surgery giving an erroneous belief of complete mesh explantation. Any following attempt becomes more difficult due to mesh fragmentation. Small infected mesh fragments are particularly challenging. Another problem is related to the close interconnection of the infected implant to the visceral organs which carries a risk of their injury during mesh explantation [12]. It is impossible to declare the true cause of the sigmoid fistula in the presented case since it appeared on the 10^th^ day postoperatively. Infection, the colon wall traumatization during mesh separation and colon localization out of an abdominal cavity in an open wound all play a role in fistula formation [13]. An enteroatmospheric fistula in an open wound is a challenging task for a surgeon and significant trouble for the patient with a harmful effect on life quality. An attempt to resolve that complication as soon as possible, in a dubious manner, resulted in a life-threatening complication for the case patient. The patient was under a real threat of death when the surgery of wound closure by flaps failed. Therefore, the local status of the wound was casted back to the initial point thus prolonging the healing for several months.

Despite the gross fecal contamination, the patient`s wound cleaned and healed spontaneously without any intervention from outside.

## 4 Conclusion

A surgical strategy varying between error and accuracy can catalyze a chain reaction of complications and surgical errors, finally resulting in life-threatening complications.

## Conflicts of interest

Nothing to disclose

## Sources of funding

This research did not receive any specific grant from funding agencies in the public, commercial, or not-for-profit sectors.

## Ethical approval

This study is exempt from ethical approval in our institution

## Consent

Written informed consent was obtained from the patient for publication of this case report and accompanying images. A copy of the written consent is available for review by the Editor-in-Chief of this journal on request.

## Author contribution

Arnolds Jezupovs: Study concept, literature review, writing the paper.

## Registration of research studies

This study is a case report

## Guarantor

Arnolds Jezupovs Head of Surgical infections clinic

## Provenance and peer review

Not commissioned, externally peer-reviewed
